# Community-based behavior change promoting child health care: a response to socio-economic disparity

**DOI:** 10.1186/s41043-016-0048-y

**Published:** 2016-04-21

**Authors:** Naoko Horii, Oumarou Habi, Alio Dangana, Abdou Maina, Souleymane Alzouma, Yves Charbit

**Affiliations:** 1 Independent consultant in Behavior change communication, Maternal child health and nutrition, Paris, France; 2 Census mapping division, National Institute of Statistics, Niamey, Niger; 3 Centre Population & Développement, Paris Descartes University, Paris, France

**Keywords:** Breastfeeding, Behavior change, Neonatal care, Vulnerability

## Abstract

**Background:**

Early initiation of breastfeeding after birth is a key behavioral health factor known to decrease neonatal mortality risks. Yet, few demographic studies examined how a community-based intervention impacts postpartum breastfeeding among the socio-economically deprived population in Sub-Saharan Africa. A post-intervention evaluation was conducted in 2011 to measure the effect of a UNICEF-led behavior change communication program promoting child health care in rural Niger.

**Methods:**

A quantitative survey is based on a post hoc constitution of two groups of a study sample, exposed and unexposed households. The sample includes women aged 15–49 years, having at least one child less than 24 months born with vaginal delivery. Rate ratio for bivariate analysis and multivariate logistic regression were applied for statistical analysis. The outcome variable is the initiation of breastfeeding within the first hour of birth. Independent variables include other behavioral outcome variables, different types of communication actions, and socio-demographic and economic status of mothers.

**Results:**

The gaps in socio-economic vulnerability between the exposed and unexposed groups imply that mothers deprived from accessing basic health services and hygiene facilities are likely to be excluded from the communication actions. Mothers who practiced hand washing and used a traditional latrine showed 2.0 times more likely to initiate early breastfeeding compared to those who did not (95 % CI 1.4–2.7; 1.3–3.1). *Home visits by community volunteers* was not significant (AOR 1.2; 95 % CI 0.9–1.5). Mothers who got actively involved in exclusive breastfeeding promotion as peers were more likely to initiate breastfeeding within the first hour of birth (AOR 2.0; 95 % CI 1.4–2.9).

**Conclusions:**

A multi-sectorial approach combining hygiene practices and optimal breastfeeding promotion led to supporting early initiation of breastfeeding. A peer promotion of child health care suggests a model of behavior change communication strategy as a response to socio-economic disparity.

## Background

Since breastfeeding is a common practice among women in Sub-Saharan Africa, a principal cause of high risks of neonatal mortality in this region was thought to be delivery complication [1–3]. However, recent studies on neonatal care interventions demonstrated that most neonatal deaths are caused by “behaviorally modifiable” factors [4]. According to the World Health Organization (WHO), early initiation of breastfeeding within 1 hour of birth “protects the newborn from acquiring infection and reduces newborn mortality” [5, 6]. The first milk produced from the sixth month of pregnancy, called colostrum, is an important source of nutrition and immune protection for the newborn [6, 7]. The immediate start of breastfeeding after birth decreases risks of maternal and neonatal mortality and long term illnesses [5, 8].

Behavior change to improve neonatal health care is one of the top priorities of action-oriented research [9]. However, we do not know much about which types of behavior change program positively impacts breastfeeding promotion. The 50 % global decline of child mortality in the last decade is mostly attributed to successful preventive and outreach delivery of oral rehydration salts and tetanus and measle vaccines. These interventions had a direct impact on child mortality reduction [10]. However, further reduction of neonatal mortality requires family- and community-based approaches aimed at improving perinatal health care and child under-nutrition [11, 12].

The Ministry of Health in Niger launched a community-based participatory communication program promoting child health care in 2008. It endorsed a priority strategic plan of action in collaboration with UNICEF to promote family- and community-based maternal and child health care. The interventions include eight key family practices (KFP):

- Exclusive breastfeeding until 6 months of age and early initiation of breastfeeding within the first hour of birth;
- Use of oral rehydration salts (ORS) for management of diarrhea within a household;
- Washing hands with soap at critical moments during the day;
- Use of insecticide-treated bednets by pregnant women and children under 5 years of age for malaria prevention;
- Introduction of timely and quality complementary feeding of children after 6 months of age;
- Use of preventive health services such as vaccination, deworming, and supplementation of vitamin A;
- Appropriate care-seeking behavior for treatment of child illnesses including malaria, diarrhea, and pneumonia;
- Birth spacing intervals of at least 24 months.

Behavior change is not attainable only by improving people’s knowledge about child health care [13–15]. Rather, behavior change communication strategies draw on dynamic processes of changing behavior to adopt a new practice [16, 17]. The communication actions implemented led people to get actively involved in the decision-making processes. As a result, expected outcomes are not only people’s improved knowledge but also their ownership of change [18]. This paper investigates the effects of different types of communication actions on initiation of breastfeeding within the first hour of birth among the most deprived group of population.

## Methods

### Study design

This is a cross-sectional study conducted in 2010 to evaluate the effects of the behavior change communication program promoting KFP in rural areas of two regions of Niger: Maradi and Zinder. Initially, the KFP intervention sites were purposively selected per administrative unit based on boundaries between villages. While there was no randomization of groups prior to the interventions, post hoc constitution of a sample of exposed and non-exposed households was applied. Hence, it is not a randomized control trial. In order to measure the change of health behavioral outcomes related to neonatal and infant health care and feeding that occurred after the program implementation, two groups were constituted in reference to exposure to the communication program. The first group exposed to the communication actions was extracted from the above intervention sites, and the second group not exposed to such interventions was selected from the villages not covered by the communication program. The survey provides information to compare the outcomes of the population exposed to the interventions and those not exposed to such interventions in a retrospective manner.

### Sampling method of the original dataset and study population

The exposed group includes 50 villages covered by the communication program in Maradi and Zinder. The unexposed group includes 25 villages in Maradi and Zinder and 52 villages in Tillabéry and Tahoua not covered by the program interventions at the time of data collection. To ensure accuracy and representativeness of the study population within each of the four regions and the program settings, the villages, households, and mothers to be interviewed were selected by random stratified sampling. The selection of villages was processed in reference to the list of all villages targeted by the program interventions in Maradi and Zinder. The number of selected households from each village was fixed at 15. At primary and secondary units of strata, the ratio of intervention and unexposed groups is 2 to 1 in Maradi and Zinder.

The original dataset of the post-intervention survey is comprised of three surveys: individual women’s survey, household survey, and community survey. Data collection using three different questionnaires for each survey was conducted in parallel in the same villages. A sample for each survey was selected based on inclusion criteria determined as follows:

#### Individual women’s survey

Women aged 15–49 years who have at least a child less than 5 years old or who are pregnant with or without a child less than 5 years old at the time of interview. Those who are not mothers but look after a child less than 5 years old as a caregiver are also included.

#### Household survey

Heads of household having a child less than 5 years old to be interviewed for collecting socio-demographic status of all household members.

#### Community survey

Representatives at community level, such as village chiefs or health workers at district health center were selected to conduct key informant interviews. All 127 villages selected for this survey filled out the questionnaire.

The study population for the secondary analysis was extracted from the original dataset of the post-intervention survey based on the following eligibility: female household members aged 15–49 years who have at least one 0- to 23-month-old child, currently pregnant or not. Confounding effects related to delivery with a caesarian section were not controlled since this variable was not included in the questionnaire. The total number of the eligible study population after cleaning the merged dataset is 2091.

### Description of the interventions

The interventions for behavior change are based on the following methods: “advocacy,” “social mobilization,” “interpersonal communication,” and “community led social change.” These strategies encourage actors to be actively involved in prompting child health care at individual, family, and community levels [19]. Based on the communication strategic conceptual framework, the relevant communication actions below were designed and implemented in Niger:

(i) Mass-media through radio program broadcasting KFP; events open to the public led by community radio stations including quiz competitions and composition of songs conveying messages related to KFP; TV clips promoting KFP; and mobile cinema led by a local NGO to show a movie addressing KFP;
(ii) Home visits by community volunteers, individual counseling during antenatal care by government health workers trained and supported by the program; and
(iii) Peer education by mothers committed to promoting KFP to other women in the neighborhood; award of best mothers who breastfed exclusively at village assemblies to select community volunteers, how to make an action plan, and community dialogue and forums mobilizing opinion leaders to discuss and promote KFP.

The participatory action research focuses on capacity building of actors outside the conventional health system, such as community volunteers. A village council chaired by the village chief selects the community volunteers. All selected volunteers follow prior to their assignment an initial training for 2 weeks on maternal and child care, communication theory and skills, individual counseling methods, and monitoring. They are not remunerated for their health promotion activities. However, the program provides them with a few incentives such as food crops, special costumes to show they are qualified community volunteers, and follow-up training and meeting sessions under the supervision of a coordinator, assigned by the program at commune and department levels, who oversees community volunteers’ performance on the ground. All women having a child less than 5 years as a mother or caregiver in the village covered by the program are to receive at least one visit at their residence per month. Each volunteer is assigned 30 and 40 households to cover. Community volunteers monitor progress of their home visits through collecting and analyzing information about behavioral outcomes of every KFP they talk about individually with mothers at the time of home visit. They are equipped with a flip chart with visual images illustrating what each KFP represents. Community volunteers who are birth attendants and/or midwives assist with delivery at health centers to fill gaps in human resources in the government health system.

### Data collection and analysis

Sixty-four enumerators were recruited and trained by the INS of Niger. Overall, 16 teams were established, each one supervisor, three enumerators, and one driver. Two supervisors were also appointed to ensure the accuracy of data collection by detecting errors in measurement and in filling out questionnaires. The data collection was processed without double entry, but data was verified and the supervisory team did consistency checks. The interviews were carried out through home visits for the individual and household questionnaires. The questionnaires formulated in French were translated into two local languages: Hausa and Zarma. Hausa was used for the interviews in Maradi and Zinder and Zarma in Tillabéry and Tahoua. Follow-up visits were done when women were absent at the time of the first visit. Data collection for all three questionnaires was conducted during a period not exceeding 20 days.

All answer sheets of questionnaires filled out by enumerators were put together once data collection was completed on the ground. Data entry was processed with CsPRO by ten trained agents [20]. The dataset was transferred to SPSS for further data management and preliminary data analysis. The original datasets in the SPSS package were converted to STATA to perform statistical analysis. Prior to the statistical tests, dataset files of an individual women’s survey and a household survey were merged into one single dataset by matching the number of households. A community survey was merged into the above combined dataset file by the name of the village.

We performed data management and statistical tests with STATA SE/13.1. The statistical models used for the secondary analysis are chi-square tests and multivariate logistic regression. Confounding effects of socio-economic status of mothers were measured based on a set of selected indicators of vulnerability measurement. Multivariate analysis only includes the variables shown to have statistically significant influence on early initiation of breastfeeding in bivariate analysis. It calculated the odds of early initiation of breastfeeding in relation to the communication program activities to which the exposed group of mothers were exposed.

### Definition of socio-economic vulnerability

To define clearly the concept of “vulnerability” employed in this research, we refer to a number of existing methods to measure poverty. In 2008, UNICEF conducted a household survey to assess the risk of children exposed to poverty-related threats [21]. This research applies the concept of relative poverty that entails inadequate and insufficient resources as described by five essential individual, family, and community levels’ livelihoods [22].

### Study limits

Cross-sectional study does not provide alternative solutions to a number of statistical biases. First, measurement bias with uncontrolled confounding effects over time is a major shortcoming. Causal relationships between outcome and independent variables cannot be determined in an accurate manner due to the communication program being implemented over a long period of time. A recall bias of the respondents is unavoidable in this type of retrospective study. At the time of interviews with mothers of children less than 24 months, they were asked to recall how soon they put their child to the breast after delivery, which is an event that occurred nearly 2 years ago for some mothers. Respondent bias might trigger errors in coding accurate information, as it is difficult for mothers to know the exact timing of giving the first breast milk by the hour [18].

### Ethics approval

We obtained the informed verbal consent of women of whom 100 % consented to participate in this study. For the surveys conducted in collaboration with UNICEF, the executing agency, the National Institute of Statistics of Niger (INS), following the Regulation no. 2004-011 dated on 30 March 2004, was exempt from requesting an ethical approval for surveys that concern human subjects with no serological or biological test such as blood tests. The INS was compelled to ask for ethics approval within the framework of the Demographic and Health Surveys (DHS-MICS) in 1998 and 2006 that included hemoglobin blood tests and verbal autopsy to identify causes of mortality. The authors of this research paper have no competing interests involved.

## Results

### Patterns of postpartum breastfeeding

When comparing the proportion of early initiation of breastfeeding between the group exposed and that not exposed to the interventions, mothers exposed to the interventions were 2.2 times more likely to initiate breastfeeding within the first hour of birth (*n* = 394, 89 %) than those not exposed (*n* = 348, 40 %) (95 % CI: 1.9-2.6) [23]. We examined each type of communication action to measure its influence on postpartum breastfeeding practices. We refer to infant feeding patterns in the last 24 h prior to the interview date among mothers of children below 6 months^1^ in Maradi, Zinder, Tillabéry, and Tahoua. Half of mothers (*n* = 216, 59 %) reported having given nothing but breast milk. Water feeding and juice consumption are the most dominant factors inhibiting early and exclusive initiation of breastfeeding: one quarter of infants fed with juice or other liquid (*n* = 95, 26 %) and 7.4 % with water (*n* = 27). According to the 2012 Niger Demographic and Health Survey, half of mothers (50.3 %) abandoned exclusive breastfeeding within the first 3 days of birth [24].

### Integrated approach of the communication program and KFP-related behavioral indicators

We calculated crude rate ratios and adjusted odds of early initiation of breastfeeding according to each type of communication program activity after having controlled for socio-economic vulnerability measured by a selected set of indicators [25]. Table 1 describes health-seeking behavior for preventive and curative child health care in relation to exposure to the communication program interventions. The key family practices (KFP)-related indicators include the following: *Use of health facility for preventive care* measured by the number of antenatal care (ANC) visits and *hand washing with soap*. An indicator, *types of toilet facilities* is also included to measure hygiene practice. Mothers exposed to the interventions were 1.7 times more likely to have *discussed with husband or grandmother about KFP* (95 % CI 1.5–1.9). The rate of *more than 4 ANC visits* as recommended by the World Health Organization [26] was 1.3 times as high as those in the unexposed group (95 % CI 1.1–1.4). Those in the exposed group had 1.6 times the rate of ANC provided by midwives compared to those who are in the unexposed group. The probability of hand washing (*n* = 557, 84 %) among those exposed to the interventions was 1.2 times that of the unexposed group (95 % CI 1.1–1.4). Mothers in the exposed group were 1.9 times more likely to use *piped water source* (*n* = 444, 66 %) compared to those in the unexposed group (95 % CI 1.6–2.1). The rate of *use of traditional latrines* reached 20 % (*n* = 132) in the exposed group, 2.2 times as high as that of the unexposed group (95 % CI 1.7–2.8). A majority of mothers in the exposed group had *no toilet facility* (*n* = 1242, 89 %); however, they were 0.82 times as likely to do outdoor defecation compared to those in the unexposed group (*n* = 487, 72 %).

**Table 1:** Key family practices of the mothers with under 24-month children in four regions of Niger (*n* = 2091)

Variables	Exposed group		Unexposed group			
	*n*	Rate	*n*	Rate	Rate ratio	95 % CI
Discussed with husband and/or mother about KFP^a^	518	0.77	633	0.45	1.72	1.53–1.93
Number of antenatal care visits	455	0.68	756	0.54		
Never	20	0.03	101	0.07		
1–3 times	192	0.29	533	0.38		
>4 times	455	0.68	756	0.54	1.25	1.11–1.41
Personal at antenatal care by						
Midwives	382	0.59	441	0.37	1.59	1.38–1.83
Doctor/nurse	268	0.41	755	0.63		
Washing hands with soap at critical moments	557	0.84	831	0.69	1.22	1.10–1.36
Source of drinking water						
Well	230	0.34	839	0.59		
Piped or public tap	444	0.66	500	0.35	1.87	1.64–2.13
Type of toilet facility						
No facility	487	0.72	1242	0.89	0.82	0.73–0.91
Traditional latrine	132	0.20	126	0.09	2.18	1.69–2.80
Ventilated/flush	54	0.08	32	0.02		

^a^Key Family Practices

Multivariate analysis measured how other KFP behavioral outcome variables affect the timing of initiating breastfeeding after birth adjusted for socio-economic status of mothers (Table 2). The odds of early breastfeeding among the mothers who discussed KFP with their family were significantly 1.6 times that of those who never discussed (95 % CI 1.2–2.1). *More than 4 ANC visits* was not associated with the timing of initiation of breastfeeding; rather, it indicates an adverse effect: those who did more than 4 ANC were 0.9 times more likely to initiate breastfeeding within the first hour of birth than those who have never been to ANC (95 % CI 0.5–1.8). Those who consulted midwives at ANC were 1.5 times more likely to practice early breastfeeding compared to those who have been received by doctors or nurses (95 % CI 1.1–2.0). Hand washing with soap and use of traditional latrines had two times the odds of initiation of breastfeeding in the first hour of birth (95 % CI 1.5–2.7 and 1.2–3.1, respectively).

**Table 2:** Effect of the communication program on early initiation of breastfeeding and KFP-related indicators (*n* = 2091)

Variables	Initiation of breastfeeding 1H of birth (adjusted OR^a^)	95 % CI	*p* value
Discussed with family members about KFP^b^			
Never	1	–	–
At least once	1.61	1.21–2.14	0.001
Antenatal care (ANC)		-	-
Never	1	-	-
1–3 times	0.69	0.36–1.35	0.28
>4 times	0.92	0.48–1.76	0.8
Type of personal at ANC			
Doctors/nurses	1	–	–
Midwives	1.49	1.09–2.04	0.01
Hand washing with soap			
No	1	–	–
Yes	1.99	1.45–2.73	<0.001
Type of toilet facility			
No facility	1	–	–
Traditional latrines	1.98	1.27–3.09	0.002
Ventilated/flush	2.91	1.21–7.02	0.02

^a^Adjusted odds ratio are calculated by controlling confounding effects of the following variables: educational attainment, age, occupation, IGA, source of drinking water, type of toilet facility, radio listening, and program interventions exposure

^b^Key family practices

### Typology of communication strategies for behavior change

First, we measured crude associations between the communication program exposure and inclusion of mothers in different types of communication actions (Table 3). *Number of attended communication actions* measured the coverage of the integrated communication program activities in which a mother participated. Overall, the mothers in the exposed group were 1.8 times more likely to participate in more than 4 communication actions (*n* = 247, 39 %) compared to those in the unexposed group (95 % CI 1.5–2.2). The rate of *KFP radio program* in the exposed group (*n* = 311, 46 %) was 1.5 times higher than that of the unexposed group (95 % CI 1.3–1.8). The probability of mothers’ husbands participating in *NGO-led events* among those exposed to the interventions (*n* = 281, 52 %) was 1.6 times that of the unexposed group (95 % CI 1.4–1.9). Those in the exposed group were 1.2 times more likely to have *KFP counseling* during ANC (95 % CI 1.1–1.4) and 1.8 times more likely to receive *home visits by community volunteers* (95 % CI 1.5–2.6) compared to those in the unexposed group. Those in the exposed group had 2.3 times the rate of getting actively involved in exclusive breastfeeding promotion (95 % CI 1.8–2.8).

**Table 3:** Communication actions implemented in four regions of Niger (*n* = 2091)

Variables	Exposed group		Unexposed group			
	*n*	Rate	*n*	Rate	Rate ratio	95 % CI
Number of attended communication actions^a^						
4 or more	247	0.39	268	0.21	1.82	1.52–2.18
1–3	371	0.58	763	0.61		
Not at all	19	0.03	230	0.18		
Listening to KFP radio program (at least once per week)	311	0.46	124	0.30	1.54	1.33–1.79
Participation at NGO events in the last month by:						
Mothers	307	0.46	422	0.30	1.53	1.32–1.78
Husband or other family member	281	0.52	345	0.32	1.64	1.40–1.93
Counseling addressing KFP at ANC (the last month)	407	0.61	690	0.49	1.23	1.08–1.39
Home visits by community volunteers	367	0.55	431	0.30	1.79	1.55–2.06
Mothers involved in promoting EB^b^	181	0.27	167	0.12	2.28	1.83–2.83

^a^Other KFP include diarrhea treatment with ORS, identification of danger signs of infant illnesses, vaccination/deworming, and birth spacing

^b^Exclusive breastfeeding

Multivariate analysis was conducted to examine the influence of each communication action on early initiation of breastfeeding adjusted for socio-economic status of mothers (Table 4). Mothers who *listened to KFP radio program* at least once in the week following the interview had 1.4 times the odds of early breastfeeding (95 % CI 1.0–1.8) compared to those who have never listened to the KFP program. Those who have received at least one of the key family practices-related counseling during the ANC visits over the month were 1.5 times more likely to initiate breastfeeding within the first hour of birth (95 % CI 1.2–2.0). The odds of early breastfeeding among those who actively promoted exclusive breastfeeding as peers was two times (95 % CI 1.4–2.9) that of those who never got involved in any peer promotion activity. However, among those who received home visits by community volunteers in the last 2 weeks, the probability of early breastfeeding was not significant (AOR 1.2, 95 % CI 0.9–1.5). In short, those who participated in more than 4 communication actions initiated breastfeeding within the first hour of birth 2.7 times more than those who have never participated in any activities (95 % CI 1.4–4.3).

**Table 4:** Effects of different types of the communication program activities on early initiation of breastfeeding (*n* = 2091)

Variables	Initiation of breastfeeding 1H of birth (adjusted OR^a^)	95 % CI	*p* value
Listening to KFP radio program			
Never	1	–	–
At least once per week	1.35	1.02–1.77	0.03
Husband’s participation at local debates about KFP		–	–
No	1	–	–
At least once during the last month	0.76	0.54–1.07	0.1
Mothers’ husbands participating at NGO events			
No	1	–	–
At least once during the last month	0.83	0.61–1.13	0.02
KFP counseling at ANC			
No	1	–	–
At least once during the last month	1.5	1.16–1.95	0.002
Home visits by community volunteers			
No	1	–	–
At least once in the last 2 weeks	1.17	0.90–1.54	0.24
Mothers involved in promoting:			
No activity	1	–	–
Exclusive BF as peer	2.0	1.39–2.89	<0.001
Promoting other KFP^b^	1.7	1.27–2.27	<0.001
Number of attended communication actions^c^			
Not at all	1	–	–
1–3	2.84	1.81–4.44	<0.001
4 or more	2.65	1.63–4.32	<0.001

^a^Odds ratio was calculated by controlling the confounding effects of socio-economic vulnerability of mothers: marital status, age of mothers, occupation, level of education, income generating activities, type of toilet

^b^Other KFP include diarrhea treatment with ORS, identification of danger signs of infant illnesses, vaccination/deworming, and birth spacing

^c^Communication actions include listening to KFP radio program, mothers’ participation at NGO events, KFP counseling at ANC, home visits, and mothers promoting KFP

## Discussion

A major limitation of this retrospective evaluation study is that no firm causal relationship can be established between the health behavioral outcome, early initiation of breastfeeding, and the identified independent variables describing communication program activities. The study design of this evaluation comparing the group exposed to the communication program with the unexposed group does not allow us to dissociate the effects of confounding factors which may alter mothers’ behavior in postpartum breastfeeding. Hence, we should carefully interpret the findings as we cannot draw definitive conclusions about why there was change in behavioral outcomes during the 2 years following the launch of the communication program. Besides, no baseline study was conducted prior to the launch of this communication program. Keeping this in mind, we deem it important to exploit the database of this evaluation study to reveal clues to understand what works and what does not work in child health care promotion.

Postpartum suboptimal breastfeeding is clearly a major public health problem in rural Niger. Twenty-four-hour recall shows that nearly half of mothers stopped breastfeeding their child exclusively before 6 months of age. It was implied that most of these mothers feed their child with other liquids than their breast milk after birth. Mothers who put their child to the breast within the first hour of birth are de facto more likely to sustain their optimal breastfeeding practice.

It is surprising to see nearly half of mothers in the unexposed group talk about KFP probably due to the contaminating effects of the program exposure (Table 1). Hygiene practices show important gaps between the two groups and reveal that mothers do not live in similar hygiene environments. It is difficult to conclude, without a baseline study conducted prior to the communication program, whether the gaps in socio-economic vulnerability of mothers between the two groups were triggered by the program interventions. The inequality in accessing hygiene infrastructure is unlikely to change in such a short time frame. It is known that behavior change communication excludes the most deprived populations without a specific action plan to identify and reach out to this vulnerable group [15]. In-depth analysis of socio-demographic characteristics of mothers over time between 2006 and 2012 in Niger suggests that those exposed to this communication program are better off than those who have not been covered by the interventions [23].

Dialogue with the husband or the child’s grandmother about KFP is one of the critical child health care-related behaviors leading all mothers including the most deprived to adopt optimal postpartum breastfeeding (Table 2). The number of mothers who had *more than 4 ANC visits* has dramatically increased in the last 5 years [24]. Midwives at government health centers trained and supported by the program were empowered and established a trustworthy relationship with pregnant women in villages and became a key communication channel for behavior change of mothers in perinatal health care as shown by the high probability of early breastfeeding among those having consulted midwives at ANC. Another important factor playing a key role to promote early initiation of breastfeeding is the child’s grandmother. They often have cumulative roles as an untrained traditional birth attendant in their village and are the first contacts for mothers to discuss newborn care. Intra-family and community interactions are a cornerstone of child health care during the perinatal period and have already shown positive influence on the early initiation of breastfeeding in previous studies [27, 28]. Nevertheless, the communication program does not implement specific actions to approach grandmothers. The evaluation study of this program does not include specific variables to assess the influence of grandmothers or traditional birth attendants on mothers’ perinatal care. Postpartum breastfeeding is determined by who is present and by what could be done at the time of delivery [29]. According to the 2012 Niger DHS, most deliver at home (*n* = 2633, 75 %) with assistance of family members or neighbors in the village who determine mothers’ postpartum breastfeeding. It is crucial to examine the role of traditional birth attendants whose share of delivery attendance represents 32.3 % (*n* = 1143) in rural Niger [24].

The findings show that regardless of socio-economic status of mothers, the chance of early initiation of breastfeeding among those who have adopted the practices described in Table 2 is greater in a multi-sectorial approach than an approach promoting a single child health care practice. UNICEF supported community-based hygiene infrastructure such as construction of drinking water pipes and traditional latrines, combined with behavior change in hygiene practices encouraging villagers to stop defecating outside. The hygiene and water sanitation program covering the same villages as the KFP program sites includes a behavior change component promoting not defecating outside, hand washing after defecation, and giving nothing but breast milk to a child in the first 6 months of birth. Hence, we draw assumptions that increased use of toilets and safe drinking water interacted positively with early initiation of breastfeeding within an intensive integrated program of environmental, maternal, and child health. In short, by addressing multiple dimensions of preventive and curative child health care, the communication program further increased early initiation of breastfeeding instead of focusing on promoting early breastfeeding.^2^ The multi-sectorial approach demonstrates change in optimal postpartum breastfeeding among the deprived group of mothers. Based on a life cycle approach to maternal and child health to address multiple dimensions of child illnesses, the communication program positively influenced postpartum breastfeeding [30].

The coverage of the program is not optimal, and some communication actions record a moderate participation (Table 3). While the majority of mothers were reached by at least one type of communication action, those having attended four or more activities include only 39 % (*n* = 515) in the exposed group. Mixing different types of behavior change strategies was thought to empower different communication channels combining both community actors and health professionals whereas the findings suggest that the extent to which mothers initiated breastfeeding according to their exposure to each type of communication action varies.

The findings of multivariate analysis on early breastfeeding according to different types of communication strategies suggest which actions change behavior in postpartum breastfeeding among the most deprived mothers in rural Niger (Table 4). Participatory community-led social change, measured by whether mothers were involved in promoting exclusive breastfeeding as peers, seems to have the most significant impact compared to other behavior change strategies in early breastfeeding promotion. The interpersonal communication defined as *home visits led by community volunteers* was no longer associated with early initiation of breastfeeding. The roles of community volunteers are worth extensive review to investigate their performances at the time of home visits. Further research is necessary to identify who are excluded from the home visits in terms of social determinants such as number of children, type of occupation, type of toilet facilities, and water sources. Community volunteers are themselves young mothers having household duties and income-generating activity. They reported having felt overwhelmed by the excessive number of households to visit. Those who live outside the catchment area are more likely to be excluded from the communication actions: they are deprived from access to health care services and basic hygiene facility around which communication actions are undertaken.

A previous statistical analysis showed socio-economic vulnerability to be a major risk factor hindering early initiation of breastfeeding in rural Niger [23]. If behavior change communication program interventions focus on better off in adopting optimal postpartum breastfeeding which is very likely to occur, there will be further gaps in newborn care for neonatal survival and health between better off and poor. We deem it important to examine whether communication program interventions are not only effective in terms of the overall number of people covered but also responsive to socio-economic disparity in promoting early initiation of breastfeeding. Poverty is beyond the scope of the BCC strategy, and the design of such interventions does not systematically prioritize actions on high-risk groups of population. Promotion of maternal and child health care was not aimed at improving living standards of mothers. Since 2012, UNICEF started testing a new framework of strategic plan of BCC program promoting maternal and child health in Western and Central Africa with a focus on vulnerable groups.^3^

Active involvement of mothers as peer promoters of KFP leads mothers to being a member of the society she belongs to. This communication action impacts the relation between mothers, their family, and community members and changes the perception of the population, encouraging them to find alternative solutions together to overcome risk factors hindering early breastfeeding practice. Groups of mothers actively involved in promoting early and exclusive breastfeeding create a dynamic change in social values [13, 31] recognized as a “good mother.” New practices therefore become acceptable when addressed to a group of mothers [31, 32] than when individually approaching a mother isolated from other women. Another implication of this participatory communication action is ownership of mothers. Active participation of mothers in planning, implementing, and analyzing the results of their actions [14, 22] positively impacted early breastfeeding beyond the fact that they are poor or better off.

## Conclusions

The study findings suggest that participation in communication actions is impaired by socio-economic vulnerability in rural Niger. Exclusion of the most deprived group of mothers from the implemented communication actions is subject to further investigation. A multi-sectorial approach addressing various dimensions of maternal and child health care, particularly hygiene practices and environmental health, positively impacts early initiation of breastfeeding as a response to socio-economic disparity. As to the typology analysis of the BCC strategies, participatory community-led social change through peer promotion of child health care suggests a response to socio-economic disparity in promoting early initiation of breastfeeding.

## Footnotes

1 According to WHO/UNICEF, complementary feeding, giving semi-solid food to supplement the required energy and nutrition provided by continued breastfeeding, should be introduced from the sixth month of birth.

2 A chi-square test using the 2012 Niger DHS dataset shows significant associations between the number of ANC visits and mothers sleeping under bednets in the previous night. Among mothers doing more than 4 ANC visits, the proportion of those sleeping under bednets (*n* = 527, 35 %) increased by 22 % compared to those doing less than one ANC visit (*n* = 76, 13 %) showing a statistically significance difference (*p* < 0.001).

3 The first author was involved in modeling this framework by adapting to an emergency context in Tchad to elaborate a strategic plan of BCC for the UNICEF Polio eradication program with a focus on the most vulnerable population in 2011.
